# Fluorescent Staining and Quantification of Starch Granules in Chloroplasts of Live Plant Cells Using Fluorescein

**DOI:** 10.21769/BioProtoc.5103

**Published:** 2024-11-05

**Authors:** Shintaro Ichikawa, Yutaka Kodama

**Affiliations:** 1 Center for Bioscience Research and Education, Utsunomiya University, Tochigi, Japan; 2 Graduate School of Regional Development and Creativity, Utsunomiya University, Tochigi, Japan

**Keywords:** Starch granule, Staining, Live-cell imaging, Fluorescein, Chloroplast, Confocal microscope

## Abstract

Plants use CO_2_, water, and light energy to generate carbohydrates through photosynthesis. During daytime, these carbohydrates are polymerized, leading to the accumulation of starch granules in chloroplasts. The catabolites produced by the degradation of these chloroplast starch granules are used for physiological responses and plant growth. Various staining methods, such as iodine staining, have previously been used to visualize the accumulation of chloroplast starch granules; however, these staining methods cannot be used to image live cells and/or provide confocal images with non-specific signals. In this study, we developed a new imaging method for the fluorescent observation of chloroplast starch granules in living plant cells by staining with fluorescein, a widely available fluorescent dye. This simple staining method, which involves soaking a leaf disk in staining solution, shows high specificity in confocal images. Fluorescent images of the stained tissue allow the cellular starch content of living cells to be quantified with the same level of accuracy as a conventional biochemical method (amyloglucosidase/α-amylase method). Fluorescein staining thus not only enables the easy and clear observation of chloroplast starch granules but also allows for precise quantification in living cells.

Key features

• Visualizes chloroplast starch granules stained with fluorescein in living cells.

• Requires only simple specimen preparation with no reagents needed other than the staining solution.

• Fluorescein is readily available worldwide.

• Highly specific method for identifying chloroplast starch granules in confocal images.

• Enables estimation of cellular starch content using fluorescent images.

## Graphical overview


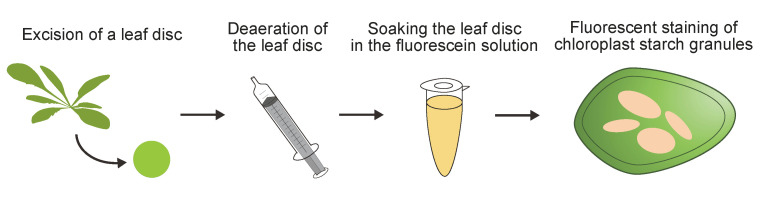


## Background

Plants perform photosynthesis during the day to convert light energy into usable chemical energy. Many plant species store this energy as polysaccharides by accumulating starch granules within chloroplasts. At night, starch granules are broken down into soluble sugars, which are then distributed to sink organs for use in growth and development [1,2]. The products mobilized from starch degradation also mediate osmotic stress tolerance [3] and facilitate stomatal opening in guard cells [4]; thus, the biosynthesis and degradation of chloroplast starch granules are crucial processes in plants.

Several staining methods have been developed for observing chloroplast starch granules in leaves, enabling the quantification and investigation of their morphology. Conventional staining methods such as iodine, toluidine blue, and periodic acid Schiff staining have been widely used [5–7]; however, these staining methods involve multiple procedures and chemical fixation, preventing rapid and/or live-cell imaging. Chloroplast starch granules have also been visualized using fluorescent staining methods, including modified pseudo-Schiff propidium iodide [4,8] and safranin O staining [9], but these methods generate non-specific signals. It is therefore necessary to develop a simple staining method that enables the observation of living cells and shows good specificity for chloroplast starch granules.

To address this need, we recently established a fluorescein staining method for visualizing chloroplast starch granules fluorescently in various living plant cells [10]. Fluorescein staining only requires the submergence of leaf tissue in staining solution for 10 min. Due to its high specificity for chloroplast starch granules, fluorescent images of stained leaf tissue can be used to quantify the cellular starch content. Fluorescein staining is therefore a valuable analytical tool for understanding chloroplast starch granules in various plant species.

## Materials and reagents

**Biological materials**

1. *Arabidopsis thaliana* accession Col-0 (Arabidopsis) (see General Note 1)

**Reagents**

1. 99% ethanol (Japan Alcohol Trading Co.)
2. Molecular sieves pack 3A (Wako, catalog number: 131-13531)
3. Fluorescein (Wako, catalog number: 065-00252)
4. Dimethyl sulfoxide (Wako, catalog number: 048-21985)
5. Fluorescein diacetate (FDA) (TCI, catalog number: F0240) (see General Note 2)

**Solutions**

1. Fluorescein staining solution (see Recipes)
2. FDA staining solution (see Recipes)

**Recipes**

1. **Fluorescein staining solution**
  **Table:** 

Reagent	Final concentration	Amount
10 mM fluorescein in ethanol treated with molecular sieves (100% ethanol)	10 μM	1 μL
Ultrapure water	n/a	Up to 1,000 μL
  The staining solution should be prepared just before use. Molecular sieves were used as dehumidifiers for 99% ethanol following manufacturer’s instructions.
2. **FDA staining solution**
  **Table:** 

Reagent	Final concentration	Amount
10 mM FDA in dimethyl sulfoxide	10 μM	1 μL
Ultrapure water	n/a	Up to 1,000 μL
  The staining solution should be prepared just before use.

**Laboratory supplies**

1. Hole puncher (2.0 mm in diameter) (Natsume Seisakusho Co., catalog number: KN-291-2)
2. Disposable 10 mL syringe and plunger (Terumo, catalog number: SS-10SZ)
3. Slide glass (super frost) (Matsunami Glass, catalog number: S024420)
4. Cover glass, 20 × 50 mm, thickness 0.16–0.19 mm (No. 1S) [Matsunami Glass, catalog number (similar product): C025501]
5. Microtube, 1.5 mL (WATSON, catalog number: 131-7155C)

## Equipment

1. Confocal microscope (Leica Microsystems, model: Leica TCS SP8X)
2. Water-immersion lens (Leica Microsystems, model: HC PL APO 63×/1.20 W CORR CS2)

## Software and datasets

1. LAS X (Leica Microsystems)
2. Fiji/ImageJ (https://imagej.net/software/fiji/downloads)

## Procedure

- **Sample preparation and fluorescein staining**
  1. Use a hole puncher to excise a leaf disk with a diameter of 2.0 mm (Figure 1A–B) (see General Note 3).
  2. Add 2–5 mL of ultrapure water and the leaf disk to a syringe using a tweezer and insert the plunger. Be careful not to spill water from the top of the syringe.
  3. Deaerate the leaf disk in water by pulling the plunger while holding the top of the syringe (Figure 1C). Repeat this process several times to adequately deaerate the leaf disk.
  4. Transfer the deaerated leaf disk into a 1.5 mL microtube with 1 mL of 10 μM fluorescein solution using a tweezer and gently invert the tube several times to immerse the leaf disk (Figure 1D).
  5. Incubate the leaf disk for 10 min with one or two gentle inversions of the tube to sufficiently stain the leaf disk.
  6. Mount the stained leaf disk onto a slide glass without rinsing and cover it with a cover glass. Ensure the slide glass is moistened with ultrapure water before mounting the leaf disk (Figure 1E).
    **Figure 1.**
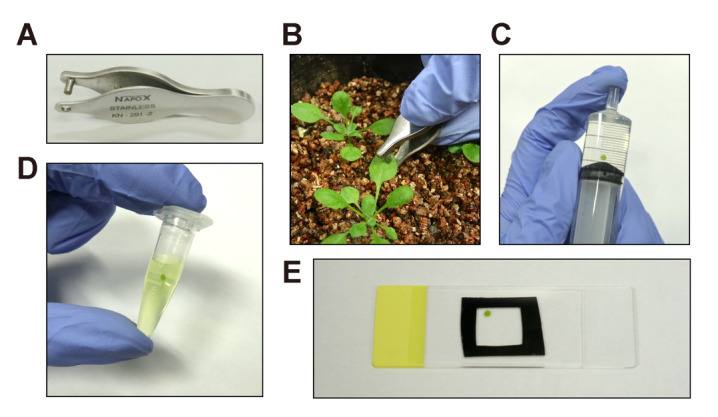
Staining and sample preparation. A. Hole puncher used to excise a leaf disk. B. Excision of a leaf disk from an Arabidopsis leaf using the hole puncher. C. Deaeration of a leaf disk using a syringe and plunger. D. Staining of a leaf disk in the fluorescein staining solution. E. Preparation of a wet-mount slide for a stained leaf disk.
- **Observation of starch granules using confocal microscopy**
  1. Place the slide on the confocal microscope and observe the fluorescein fluorescence (which represents stained starch granules) using the settings shown in Table 1 (Figure 2) (see General Note 4).
    **Table 1.** Confocal microscope (Leica TCS SP8X) settings for detection of fluorescein and chlorophyll fluorescence

	General condition for fluorescein	Chlorophyll
Light source	White light laser (WLL)	WLL
Laser intensity	21% (30% intensity from the 70% power WLL)	21%
Objective	HC PL APO 63×/1.20 W CORR CS2 (water-immersion lens)	HC PL APO 63×/1.20 W CORR CS2
Format	100 Hz/512 × 512 pixels	100 Hz/512 × 512 pixels
Detector	Hybrid detector (HyD)	HyD or photomultiplier tube (PMT)
Excitation	488 nm	488 nm
Emission range	500–550 nm	680–720 nm
Time gating	0.5–1.2 ns	Off
    **Figure 2.**
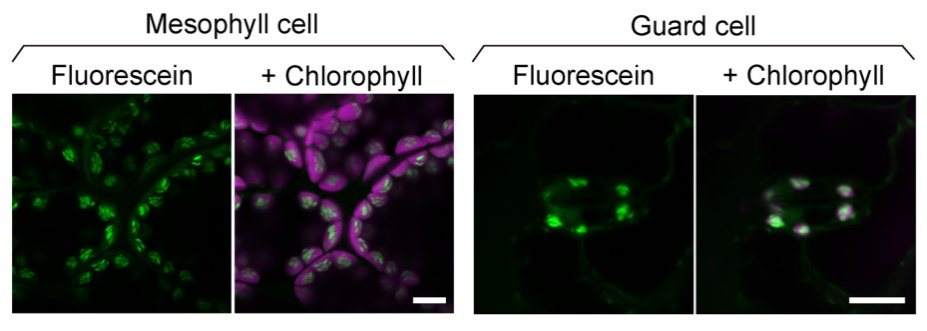
Confocal images of fluorescein-stained starch granules in mesophyll and guard cells. Leaf cells of 3-week-old Arabidopsis plants stained with 10 µM fluorescein for 10 min. The green channel displays the fluorescence of fluorescein-stained starch granules, and the magenta channel displays the fluorescence of chlorophyll. Scale bars, 10 μm.

## Data analysis

**Measurement of the area of the fluorescein signals representing the starch granules**

1. Export confocal images of fluorescein-labeled starch granules as Tagged Image File Format (TIFF) files with a scale bar using LAS X software.
2. Open the images in Fiji/ImageJ (Figure 3A).
3. Convert the image format from RGB color to 8-bit to perform the following binarizing process (*Image* > *Type* > *8-bit*).
4. Use the *Straight tool* to draw a line along the scale bar and then set the scale (*Analyze* > *Set Scale*) (Figure 3B).
5. Binarize the image by adjusting the threshold (*Image* > *Adjust* > *Threshold*) (Figure 3C). Visually adjust the threshold value until the signal area of the binarized image closely matches that of the original confocal image (see General Notes 5 and 6).
6. Use the *Wand tool* to select the binarized starch granule signals (Figure 3D).
7. Measure the area of the selected starch granule signals (*Analyze* > *Measure*) (Figure 3E) (see General Note 7).

## Validation of protocol

This protocol or parts of it were used and validated in the following research article:

- Ichikawa et al. [10]. Fluorescein staining of chloroplast starch granules in living plants. *Plant Physiology* (Staining experiments with chloroplast starch granules: Figure 1A, C, F; Figure 3; Figure 4 A; Supplementary Figure S1B, C; Supplementary Figure S2; Supplementary Figure S3; Supplementary Figure S4A; Supplementary Figure S6B; Supplementary Figure S8; Supplementary Figure S9A; and Supplementary Figure S10. Correlation analysis of the area of fluorescent starch granules in chloroplasts and the starch content in leaves: Supplementary Figure S4. Spectrum measurement: Supplementary Figure S6C).
  **Figure 3.**
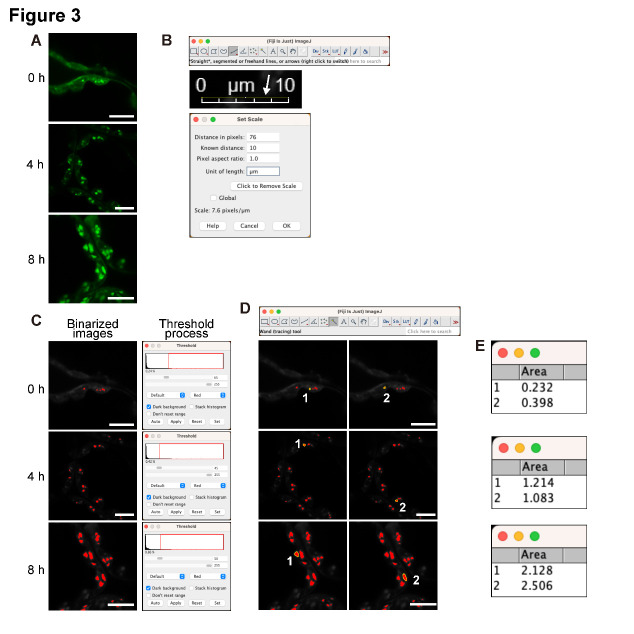
Quantification of the area of chloroplast starch granules stained with fluorescein. A. Fluorescein-stained starch granules in Arabidopsis mesophyll cells irradiated with white light (approximately 50 μmol photons m^–2^ s^–1^) for 0, 4, and 8 h after a 24 h dark treatment. B. Setting the scale using the *Straight tool* in Fiji and the bar in the TIFF image. C. Setting the threshold using 8-bit color images. D. Selecting the starch granule signals with the *Wand tool* of Fiji. E. Quantifying the area of the selected chloroplast starch granules. All scale bars, 10 μm. These quantitative data are used in Figure 5 and our previous study [10].

## General notes and troubleshooting

**General notes**

1. Applicability of fluorescein staining: Fluorescein staining can be used in many plant species including Arabidopsis, *Nicotiana benthamiana*, soybean (*Glycine max*), strawberry (*Fragaria* × *ananassa*), lettuce (*Lactuca sativa*), tomato (*Solanum lycopersicum*), cucumber (*Cucumis sativus*), and the model liverwort *Marchantia polymorpha* [10]. In our previous tests, chloroplast starch granules were successfully observed in a variety of land plants using fluorescein staining.
2. Enhanced fluorescence with FDA: The use of FDA instead of fluorescein greatly enhances the fluorescence intensity of chloroplast starch granules in living cells [10]. The recipe for FDA staining solution is noted in the Recipes section. The protocol for FDA staining is the same as that for fluorescein staining [10].
3. Leaf disk size: Any size of leaf disk can be used, but a smaller size is preferable for staining and observing leaf cells. A leaf punch is not essential.
4. Fluorescein fluorescence detection: To accurately detect fluorescein fluorescence in plant cells, we provide the spectrum of excitation and emission wavelengths of fluorescein in the starch granules (Figure 4). Although we employed the confocal microscopy system made by Leica (SP8X), confocal microscopy systems made by other manufacturers can also be used. Furthermore, starch granules stained by fluorescein could be observed using conventional fluorescence microscopy.
  **Figure 4.**
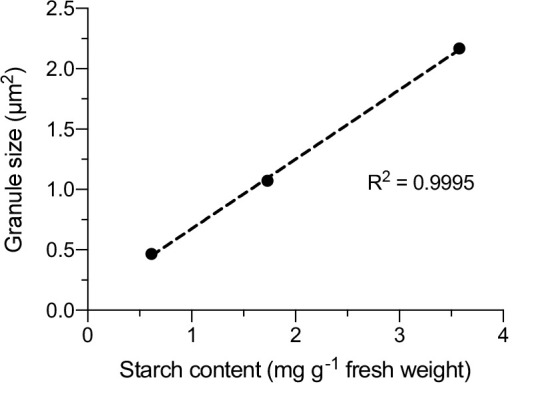
Spectrum of fluorescein in the chloroplast starch granules of living cells. Excitation and emission spectra of fluorescein adhering to the chloroplast starch granules in *Arabidopsis* mesophyll cells were measured at 5 nm intervals using xyΛ and xyλ modes, respectively, within LAS X. The measurement was repeated for 10 starch granules, and relative means are plotted. These data were used in a previous study [10].
5. Thresholding for binarization: Typically, a constant threshold value or auto-threshold is desirable for binarization [11]; however, these thresholding processes may not precisely delineate the area of the starch granules because fluorescein accumulates not only at starch granules but also in the stroma after the treatment of plant cells [10]. Fluorescence from the stroma represents a non-specific signal, so users should manually adjust the threshold value for each image to achieve optimal delineation.
6. Manual tracing options: The *Polygon selection tool* or *Freehand selection tool* within Fiji/ImageJ can also be used to determine the area of chloroplast starch granules by manual tracing, but we employed the threshold process because it is laborious to manually trace the small fluorescence signals of starch granules.
7. Correlation with starch content: There is a strong positive correlation (R^2^ = 0.9995) between the area of chloroplast starch granules and the enzymatically quantified starch amount [10]. This indicates that fluorescein signals from starch granules represent the starch content of leaves (Figure 5).
  **Figure 5.**
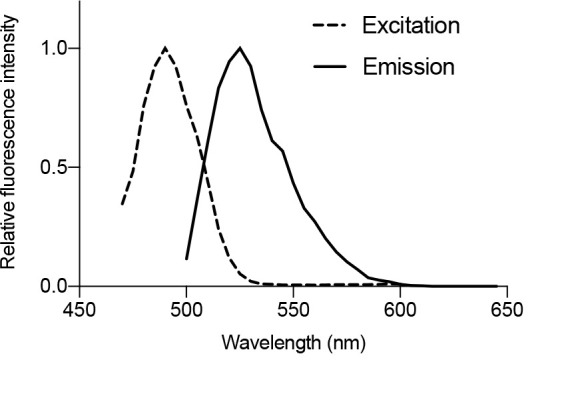
Correlation between chloroplast starch granule size and the biochemically determined starch content in Arabidopsis leaves. Plots of the starch granule size within chloroplasts (fluorescein imaging) and the leaf starch content (amyloglucosidase/α-amylase method; Millipore Sigma, STA20) at 0, 4, and 8 h of white light illumination (approximately 50 μmol photons m^–2^ s^–1^) after a 24 h dark incubation. At each time point, the size of 10 starch granules was measured, and the starch content was measured four times. Average values for both measurements were plotted. These data were used in a previous study [10].

**Troubleshooting**

Problem 1: Chloroplast starch granules are not observed using confocal microscopy.

Possible cause: Inadequate staining by fluorescein.

Solutions: Improve staining efficiency by using the fluorescein staining solution instead of water during the deaeration step. Extend the staining time; staining for less than 3 h is typically sufficient to visualize chloroplast starch granules in living plant cells [10]. Consider using FDA instead of fluorescein, as it enhances the fluorescence intensity of the chloroplast starch granules.

Problem 2: Chlorophyll autofluorescence interferes with the detection of fluorescein fluorescence.

Solutions: Use the time-gating method to exclude chlorophyll autofluorescence [12]. If time-gating is not available, ensure that the samples are well-stained with fluorescein or FDA to reduce interference. Set the excitation wavelength of fluorescein to a longer wavelength (e.g., 514 nm) to decrease chlorophyll excitation [12]. However, note that this may reduce fluorescence intensity of fluorescein due to non-optimized excitation. Bleached chlorophyll has an elevated autofluorescence [12]; therefore, lengthy irradiation of the specimen by the excitation laser should be avoided.
